# NUTRITIONAL ASSESSMENT AND HAND GRIP STRENGTH OF CANDIDATES FOR SURGERY OF THE GASTROINTESTINAL TRACT

**DOI:** 10.1590/S0102-67202014000200005

**Published:** 2014

**Authors:** Thalita Morgana Guimarães SILVEIRA, Juliana Barbosa de SOUSA, Maria Luiza Ferreira STRINGHINI, Ana Tereza Vaz de Souza FREITAS, Paulla Guimarães MELO

**Affiliations:** From the Hospital das Clínicas, Universidade Federal de Goiás (Clinics Hospital, Federal University of Goiás), Goiânia, GO, Brazil.

**Keywords:** Nutritional assessment, Malnutrition, Anthropometry, Handshake, Surgery

## Abstract

**Background:**

The assessment of nutritional status in clinical practice must be done with simple, reliable, low cost and easy performance methods. The power of handshake is recognized as a useful tool to evaluate muscle strength, and therefore, it is suggested that can detect malnutrition.

**Aim:**

To evaluate the nutritional status by subjective global assessment and power of handshake preoperatively in patients going to gastrointestinal surgeries and to compare the diagnosis obtained by subjective global assessment with traditional anthropometric methods and power of handshake.

**Methods:**

A cross-sectional study was conducted with patients for surgery in the gastrointestinal tract and related organs. Socioeconomic and anthropometric data, applied to subjective global assessment and checked the power of handshake, were collected. The force was obtained by the average of three measurements of the dominant and non-dominant hand and thus compared with reference values ​​of the population by sex and age, for the classification of nutritional risk.

**Results:**

The sample consisted of 40 patients, 24-83 years, and most women (52.5%) housewives (37,5%) and diagnosed with cancer (45%). According to subjective global assessment, 37.5% were classified as moderately malnourished; 15% were underweight by BMI measurements; 25% had arm circumference at risk for malnutrition (<percentil 5); 60% reported recent weight loss; and 37.5% low clamping force in power of handshake on non-dominant hand (left).

**Conclusion:**

A significant association was observed for the diagnosis of nutritional subjective assessment with anthropometric methods and strength of the handshake only at the non-dominant limb.

## Abstract (pt)

**Racional:**

Na prática clínica são necessários métodos simples, confiáveis, de baixo custo e de fácil acesso para a avaliação do estado nutricional. A força do aperto de mão é reconhecida como uma ferramenta útil para avaliar a força muscular, e consequentemente, elemento que pode detectar desnutrição.

**Objetivo:**

Avaliar o estado nutricional tanto pela avaliação nutricional subjetiva global como pela força do aperto de mão de pacientes em pré-operatório e comparar o diagnóstico obtido por ambas e os tradicionais métodos antropométricos.

**Métodos:**

Foi realizado estudo transversal com pacientes internados para operação em trato gastrointestinal e órgãos anexos. Foram coletados dados socioeconômicos, antropométricos, aplicado a avaliação nutricional subjetiva global e verificada a força do aperto de mão. Esta força foi obtida pela média de três medidas da mão dominante e não dominante e dessa forma, comparada com valores de referência da população brasileira, segundo sexo e idade, para a classificação do risco nutricional.

**Resultados:**

A amostra foi constituída por 40 pacientes de 24 a 83 anos, a maioria mulheres (52,5%) donas de casa (37,5%) e com diagnóstico de neoplasia (45%). Segundo a avaliação nutricional subjetiva global, 37,5% foram classificados como desnutridos moderados; 15% com baixo peso pelo IMC; 25% com circunferência braquial em risco para desnutrição (<percentil 5); 60% com perda ponderal recente; e pela força do aperto de mão, 37,5% tinham baixa força na mão não dominante (esquerda).

**Conclusão:**

Houve associação significativa do diagnóstico nutricional observado pela avaliação subjetiva com os métodos antropométricos e a força do aperto de mão apenas no membro não dominante.

## INTRODUCTION

Malnutrition is often found in the hospital environment, and is strongly associated with increased complications and cost, longer hospitalization and mortality^8^. Studies in surgical patients show its prevalence in 30-50%^³^. Given the important influence of nutritional status on clinical outcome of patients eligible for surgical procedures, every effort should be taken to identify patients at nutritional risk ^6^.

To assess the nutritional status of hospitalized patients various methods, among which dietary, anthropometric, biochemical, immunological, clinical history and physical examination, can be used^24^.

A Subjective Global Assessment (SGA) nutritional assessment from several factors, such as energy supply of macro and micronutrients, assesses the loss of weight, fat and muscle mass over a given period. In hospitalized patients, the SGA has the power not only to assess the nutritional status, but also the general health of the patient, which influences the nutritional status^23^.

Despite the importance on correlation of the muscle strength and nutritional/functional status, still there are limited methods to do it^27^. Strength of Handshake (SH) is used to measure muscle strength, which is directly related to the nutritional status of the individual. Thus, this method has been used as a diagnostic parameter of nutritional condition^19^.

Functional indicators are correlated with clinical complications and are more sensitive and relevant to observe nutritional changes in short-term. Due to quickness, no charge and noninvasive, SH is one of the leading indicators described in the literature^22^.

Reduction in muscle mass is a prognostic parameter for complications in the postoperative period and the loss of functional capacity of skeletal muscle is a predictor of morbimortality^10^. So, this study proposes to assess the nutritional status diagnosed by SGA and functional capacity through the SH patients preoperatively for operation of the digestive system and compare the diagnosis obtained by SGA with anthropometric measurements and SH.

## METHOD

Cross-sectional study conducted at Hospital das Clínicas, Federal University of Goiás, Goiânia, GO, Brazil, with adult patients admitted to carrying out operations of the digestive tract in the period October-November 2013.

Were included patients aged greater than or equal to 19 years, of both genders, with up to 48 h after admission in the preoperative period of operation of the gastrointestinal tract without edema or motor disability that prevented anthropometric assessment. They were informed about the research objectives, risks and benefits and signed a consent form. The study was approved by the Ethics and Research of the hospital with protocol number 411 495.

Was applied in the first 48 h after admission, the SGA proposed by Detsky et al^8^, modified and adopted by the Group for Enteral and Parenteral Nutritional Support, involving data on weight change, dietary intake, gastrointestinal symptoms, functional capacity and physic examination^23^. Patients were classified into three categories according to score: well nourished (≤17 points), moderately malnourished (17-22 points) and severe malnourished (≥22 points). Socioeconomic and clinical data were obtained by interview and the medical records. The variables in the questionnaire were: age at the time of the interview, marital status (with and without companion), family economic income based on minimum salary (less than one, one, two and more than three), employment status (employed , self-employed, housewife home and not working), smoking (yes, no and former), alcohol consumption (yes, no and ex) , regular physical activity (practicing and non practicing) considering practitioners as those with moderate-intensity exercise intense, lasting for at least 20 minutes three to five times the week^18^. The disease and the affected organ were collected as described in the medical records.

Usual weight, current weight, percentage weight loss (%WL), height, BMI, arm circumference (AC), triceps skinfold thickness (TSF) and arm muscle circumference (AMC) for nutritional assessment, were used.

The patient was asked about the average weight of the last six months and current weight obtained using a portable scale with a capacity of 150 kg and 0.1 kg graduation, as described by Lohman et al^17^.

The WL% was obtained by the equation: (usual weight - actual weight) / 100 x usual weight and value interpreted as proposed by Blackburn et al^5^. According to these authors, weight losses of up to 10% in six months are considered significant (%WL<10 %) and greater than 10% severe (%WL>10 %). Height in meters has been obtained by means of stadiometer coupled to platform scale.

BMI was calculated by the formula of Keys et al^15^, using the parameters from the World Health Organization^27^ for adults and Lipschitz^16^ for seniors. In this study, the nutritional status was grouped into three categories: underweight, normal weight and overweight/obesity.

For AC not extendable measuring tape, positioned at the midpoint of the right arm between the acromion of the scapula and the olecranon^17^, was used. To measure the TSF Lange Skinfold caliper Calipter was used, the final value being the average of three measurements and CMB obtained from the equation: AMC (cm) = AC (cm) - [TSF (mm) x 0.314] using the method also described by Lohman et al^17^.

For the three measurements (AC ,TSF and AMC), the percentile rankings as age and sex was established by Frisancho^11^ and nutritional diagnosis as proposed by Blackburn et al^5^ that classifies the cutoff point below the 5^th^ percentile as indicative of malnutrition.

The SH was performed on the dominant upper limb (WL gift) and nondominant (not gift SH) using the portable mechanical dynamometer Takei variation 1-100 kgf and 0.5 kgf accuracy. The measurement was performed with the patient standing, adjusted to the size of the hand, with the arm in 90° angle not supported on the abdomen, holding the dynamometer with the palm up and putting the arm in lower direction, increasing the strength; so, with the arm rectified was applied the maximum strength^14^.

Three measurements were performed with an average interval of five seconds and the average was used for analysis. Patients cutoff were classified as Schlussel et al^22^. Those with SH less than 10 percentile were classified as "low muscle strength" and those greater than that as "preserved muscle strength".

Data were tabulated using Excel 2007 (Microsoft) spreadsheet specially developed for research in double entry with the objective of minimizing errors. The statistical analysis was performed using SPSS version 16.0. Was used for categorical data descriptive statistics with measures of central tendency (mean and simple frequencies) and dispersion (standard deviation). To assess the association between variables of nutritional diagnosis, the logistic regression analysis test was used, with a significance level of 5% (p<0.05).

## RESULTS

The study involved the participation of 40 patients, 21 women and 19 men. The majority of the population was adult (60%), mean age 53.5 (±15.6) years. Among the participants 47.5% had a partner, 47.5% reported a monthly income of one minimum salary. Regarding employment status, 25% were employed, 27.5% self-employed, 37.5% housewives and 10% were not employed. All elderly responders reported being retired.

Regarding lifestyle, only 9% of participants practiced regular exercise, 15% were smokers and drinkers, 37.5 % ex-smokers and 40% ex-alcoholic.

The reported diseases were: rectal cancer (7), colon cancer (3), stomach cancer (1), esophagus cancer (2), papilla tumor (1), pancreas cancer (1), cholangiocarcinoma (2), cholelithiasis (3), incisional hernia (1), adenomatous polyposis (1) in Crohn disease (2), ulcerative colitis (1), Mirizzi (1), achalasia (5), megacolon (2), corrosive stricture of the esophagus (2), reconstruction intestinal transit (4) and splenectomy (1). Considering the location, it was observed that 50% of patients were admitted for intestinal operations, 20% esophageal, 7.5 % gastric and 22.5% gallbladder, liver, pancreas and others. Among the participants, 45% were diagnosed with cancer (Table 1).

**TABLE 1:** Demographic, socioeconomic and clinical candidates for the operations of the digestive tract (n=40)

Variables	n	%
Gender		
Female	21	52,50
Male	19	47,50
Age (years)		
24 – 59	24	60,00
60 – 83	16	40,00
Marital status		
Without partner	21	52,50
With partner	19	47,50
Income		
<1 MW*	6	15,00
1MW*	19	47,50
2MW *	8	20,00
>3MW *	7	17,50
Work situation		
Salaried	10	25,00
Autonomous	11	27,50
Housewife	15	37,50
Does not work	4	10,00
Retired (a)		
Yes	16	40,00
Not	24	60,00
Physical activity		
Does not practice	31	77,50
Practice	9	22,50
Smoker		
Yes	6	15,00
Not	19	47,50
Ex-smoker	15	37,50
Alcoholic		
Yes	6	15,00
Not	18	45,00
Ex-alcoholic	16	40,00
Organ involved by disease		
Esophagus	8	20,00
Stomach	3	7,50
Intestine	20	50,00
Adnexa	9	22,50
Neoplasia		
Yes	18	45,00
Not	22	55,00

* WM: minimum salary

The classification of nutritional status by SGA showed 62.5% of well- nourished participants and 37.5% moderately malnourished. Patients with severe malnutrition were identified. Presented average current weight 62.3 kg±.33 kg and height 1.60 m± 3.03 m, 60% reported weight loss in the past six months, and half of these had severe loss (weight loss ≥10%). Regarding BMI, 57.5% were normal weight (24.10±4.04 kg/m²), mean of 28.80±4.37 CB cm; CMB 224.95±34.74 mm and TSF 20.04±9.37 mm, 25%, 45% and 15% classified as malnourished respectively (Table 2).

**TABLE 2:** Anthropometric and physical description of the population candidate for operation of the digestive tract (n = 40)

Variables	n	%	Average	SD^8^
SGA^1^				
well nourished	25	62,5		
Moderately malnourished	15	37,5		
Usual weight (kg)	40	100,0	65,54	15,59
Current weight (kg)	40	100,0	62,30	13,33
Height (m)	40	100,0	1,60	13,03
%WL^2^ (kg)			11,54	8,06
≥10%	12	30,0		
<10%	12	30,0		
maintained weight	5	12,5		
gained weight	11	27,5		
BMI^3^ (kg/m²)			24,10	4,04
underweight	6	15,0		
eutrophic	23	57,5		
Overweight / obesity	11	27,5		
AC^4^ (cm)			28,80	4,37
< perc 5	10	25,0		
> perc 5	30	75,0		
TSF^5^ (mm)			20,04	9,37
< perc 5	6	15,0		
> perc 5	34	85,0		
AMC^6^ (mm)			224,95	34,74
< perc 5	18	45,0		
> perc 5	22	55,0		
SH^7^ dominant (kgf)			24,73	8,47
< perc10	14	35,0		
> perc10	26	65,0		
SH nondominant (Kgf)			24,21	8,78
< perc10	15	37,5		
> perc10	25	62,5		

1 ASG=subjective global assessment;

2 %WL=percentage of weight loss;

3 BMI=body mass index;

4 AC=arm circumference;

5 TST=triceps skinfold thickness;

6 AMC=arm muscle circumference;

7 SH=strength of handshake;

8 SD=standard deviation

In SH, all patients reported being right-handed, so the right hand was dominant. The average strength in the dominant hand was 24.73+/-8.47 kgf, being 35% of patients classified as having low muscle strength (WL<10 perc), similar to that found in the non-dominant hand 24,21 kgf+8,78 and 37.5% classified as having low muscle strength (Table 2) .

Associating the percentage of weight loss with SGA was observed that 86.7% of the malnourished patients had recent weight loss, and 30% of serious loss (weight loss ≥10%), p=0.005. Regarding BMI, 26.7% of moderately malnourished were underweight and 92.0% well nourished/eutrophic or with overweight/obesity, statistically significant (p=0.023) between the used methods.

Regarding the arm circumference was observed that 88% of those classified as well nourished by SGA were classified as out of nutritional risk by AC>perc 5 and also 96% of well-nourished had higher than 5^th^ percentile TSF. By analyzing the values ​​of AMC in relation to anthropometric data, it was observed that 68% of well-nourished and malnourished 33.3% had no nutritional risk (AMC>5 perc), with no statistical significance (p< 0.05) between the three methods of anthropometric assessment and subjective global assessment.

Regarding SH, no statistical significance between nutritional diagnosis established by SGA and the values ​​of SH preserved and not preserved in the dominant hand (right) was observed. Among the well-nourished, 68% were classified as preserved strength, as well as among malnourished, 60% had preserved force (SH>10 perc). On the non-dominant hand (left), only 24% well nourished had low strength and more than half (60%) of malnourished showed low SH force (<10 perc), which led to significant correlation between the methods (p=0.027) (Table 3).

**TABLE 3:** Comparison of Subjective Global Assessment (SGA) with other methods of classification of nutritional risk for candidates to operation on digestive tract (n = 40)

ASG^1^	Well nourished		Malnourished moderate		
	N= 25 (62,5%)		N= 15 (37,5%)		
	n	%	n	%	p^8^
%WL^2^					
≥10%	3	12,0	9	60,0	
<10%	8	32,0	4	26,7	0,005
maintained weight	4	16,0	1	6,7	
gained weight	10	40,0	1	6,7	
BMI^3^					
underweight	2	8,0	4	26,7	
eutrophic	13	52,0	10	66,7	0,023
overweight/obesity	10	40,0	1	6,7	
AC^4^					
<perc 5	3	12,0	7	46,7	
>perc 5	22	88,0	8	53,3	0,021
TSF^5^					
<perc 5	1	4,0	5	33,3	
>perc 5	24	96,0	10	66,7	0,032
AMC^6^					
<perc 5	8	32,0	10	66,7	
>perc 5	17	68,0	5	33,3	0,038
SH^7^ dominant					
<perc 10	8	32,0	6	40,0	
>perc 10	17	68,0	9	60,0	0,608
SH Nondominant					
<perc 10	6	24,0	9	60,0	
>perc 10	19	76,0	6	40,0	0,027

1 ASG=subjective global assessment;

2 %WL= percentage of weight loss;

3 BMI=body mass index;

4 AC= arm circumference;

5 TST=triceps skinfold thickness;

6 AMC=arm muscle circumference;

7 SH=strength of handshake;

8 p=binary logistic regression test

## DISCUSSION

Several methods of measurement are proposed to assess the nutritional status of patients. However, studies have shown the inadequacy of any single method or tool used in nutritional assessment of the patient, and therefore required the use of a combination of several methods^21^.

Multicenter studies with hospitalized patients showed malnutrition ranging between 50-88%, depending on the SGA^21,25,6^, data superior to those found in this study where better nourished patients were observed and there were no severe malnutrition.

According to the Brazilian Nutrition Examination Survey study involving 4,000 patients hospitalized in public assistance distributed in 25 hospitals in different Brazilian states, it was identified that 20.1% of hospitalized patients had cancer^25^, lower than the value observed in this study, in which 45% had cancer.

Study in the preoperative period with 80 patients with digestive tumors, 23 gastric and six pancreatic, found that 53% lost 5% of usual weight in the last three months^7^, similar to data observed in this study, in which 60% had weight loss in the past six months.

Research conducted with hospitalized patients compared methods of nutritional screening, including the SGA with SH, and noted that patients classified as malnourished by SGA had SH median of 25 kgf and well nourished median of 27.3 kgf, p=0.99. There was poor agreement with the SH, SGA, but patients considered malnourished, had a lower median SH compared to well nourished^12^, values ​​similar to this group, in which the dominant SH showed no significant correlation with SGA since 60% of malnourished presented SH preserved, with an average of 24.73 kgf dominant force.

In the studied population, the majority of patients showed eutrophy according to BMI and nondominant SH preserved, average 24.21 kgf, similar to that found by Pastore et. al^20^ who evaluated the nutritional status and SH in 77 patients with digestive and lung cancer with a mean of 63.9 years and observed 60% of normal and non-dominant SH average of 25.7 kgf. Regarding the SGA results were divergent, which may be because it is a subjective evaluation that requires experience and training interviewer^23^.

Study conducted at Hospital das Clinicas of Porto Alegre, Brazil, with 75 patients compared the nutritional status between functional methods, anthropometry and SGA in patients with Crohn's disease in clinical remission^4^ and noted that 37.3% by TSF were malnourished and 73.3% at nutritional risk by SH, superior results to those found in the present study. By AC 26.7% were malnourished, similar to that found here (25%); 9.3% malnourished by AMC, 18.7% by SGA and 6.7% by BMI, lower than those observed in this study, 45% 37.5% and 15% respectively.

Alvares da Silva and Silveira^1^ in a study of 108 healthy individuals, suggested normal parameters for grip strength, ranking as nutritional risk the ones with values ​​below normal. Nunes et al^19^ in a study with cirrhosis, the SH diagnosed 58.8% of malnourished, being the method that more identified divergent malnutrition whereas 37.5% had nutritional risk measured by the non-dominant SH.

Gottschall et al.^13^ concluded that the SH seems to be the most sensitive method for the diagnosis of malnutrition in patients with cirrhosis due to hepatitis C. According to these authors, the diagnosis of malnutrition measured by SH was superior to other methods (SGA, TSF, AMC, BMI), reflecting the ability of this method in diagnosing nutritional risk in the absence of any clinical aspect^1,13.19^. Moreover, Alvares da Silva and Silveira^2^ study with cirrhotic patients, reported that the SH has the ability to detect cases of malnutrition in up to 100 %.

## CONCLUSION

Most patients in the preoperative period of digestive surgery were well nourished, eutrophic and with SH preserved. When comparing different methods of assessment, was observed association among diagnoses of malnutrition encountered by subjective evaluation, anthropometry and non-dominant SH.
